# Syndromic and Non-Syndromic Patients with Repaired Tetralogy of Fallot: Does It Affect the Long-Term Outcome?

**DOI:** 10.3390/jcm11030850

**Published:** 2022-02-06

**Authors:** Giulio Calcagni, Camilla Calvieri, Anwar Baban, Francesco Bianco, Rosaria Barracano, Massimo Caputo, Andrea Madrigali, Stefani Silva Kikina, Marco Alfonso Perrone, Maria Cristina Digilio, Marco Pozzi, Aurelio Secinaro, Berardo Sarubbi, Lorenzo Galletti, Maria Giulia Gagliardi, Andrea de Zorzi, Fabrizio Drago, Benedetta Leonardi

**Affiliations:** 1 Department of Pediatric Cardiology and Cardiac Surgery, Bambino Gesù Children’s Hospital, IRCCS, 00146 Rome, Italy; giulio.calcagni@opbg.net (G.C.); camilla.calvieri@yahoo.it (C.C.); anwar.baban@opbg.net (A.B.); andrewmadri@gmail.com (A.M.); stefanisilva.kikina01@icatt.it (S.S.K.); marcoalfonso.perrone@opbg.net (M.A.P.); lorenzo.galletti@opbg.net (L.G.); mgiulia.gagliardi@opbg.net (M.G.G.); andrea.dezorzi@opbg.net (A.d.Z.); fabrizio.drago@opbg.net (F.D.); 2 Department of Paediatric and Congenital Cardiac Surgery and Cardiology, AOU Ospedali Riuniti Ancona “Umberto I, G. M. Lancisi, G. Salesi”, 60123 Ancona, Italy; francesco.bianco@ospedaleriuniti.marche.it (F.B.); marco.pozzi@ospedaliriuniti.marche.it (M.P.); 3 Adult Congenital Heart Disease Unit, Monaldi Hospital, 80131 Naples, Italy; rosaria.barracano@ospedalideicolli.it (R.B.); berardo.sarubbi@ospedaledeicolli.it (B.S.); 4 Bristol Heart Institute, Bristol Medical School, University of Bristol, Bristol BS2 8 HW, UK; massimo.Caputo@uhbw.nhs.uk; 5 Genetics and Rare Diseases Research Division, Bambino Gesù Children’s Hospital, IRCCS, 00146 Rome, Italy; mcristina.digilio@opbg.net; 6 Advanced Cardiothoracic Imaging Unit, Department of Imaging, Bambino Gesù Children’s Hospital, IRCCS, 00146 Rome, Italy; aurelio.secinaro@opbg.net

**Keywords:** Tetralogy of Fallot (ToF), congenital heart disease (CHD), genetic syndromes, cardiac magnetic resonance imaging (cMRI), pulmonary valve replacement (PVR)

## Abstract

Background: The impact of genetic syndromes on cardiac magnetic resonance imaging (cMRI) parameters, particularly on right and/or left ventricular dysfunction, associated with clinical parameters following the repair of Tetralogy of Fallot (rToF) is not well known. Therefore, this study aimed to assess the differences in clinical, surgical, and cMRI data in syndromic and non-syndromic rToF patients. Methods: All syndromic rToF patients undergoing a cMRI without general anesthesia between 2010 and 2020 who were able to match with non-syndromic ones for birth date, sex, type of surgery, timing of cMRI, and BSA were selected. Demographic, clinical, surgical, MRI, ECG, and Holter ECG data were collected. Results: A total of one hundred and eight rToF patients equally subdivided into syndromic and non-syndromic, aged 18.7 ± 7.3 years, were studied. Del22q11.2 and Down syndrome (DS) were the most frequent syndromes (42.6% and 31.5%, respectively). Regarding the cMRI parameters considered, left ventricular (LV) dysfunction (LVEF < 50%) was more frequently found in syndromic patients (*p* = 0.040). In addition, they were older at repair (*p* = 0.002) but underwent earlier pulmonary valve replacement (PVR) (15.9 ± 5.6 vs. 19.5 ± 6.0 years, *p* = 0.049). On multivariate Cox regression analysis, adjusted for age at first repair, LV dysfunction remained significantly more associated with DS than del22q11.2 and non-syndromic patients (HR of 5.245; 95% CI 1.709–16.100, *p* = 0.004). There were only four episodes of non-sustained ventricular tachycardia in our cohort. Conclusions: Among the cMRI parameters commonly taken into consideration in rToF patients, LV dysfunction seemed to be the only one affected by the presence of a genetic syndrome. The percentage of patients performing PVR appears to be similar in both populations, although syndromic patients were older at repair and younger at PVR. Finally, the number of arrhythmic events in rToF patients seems to be low and unaffected by chromosomal abnormalities.

## 1. Introduction

Tetralogy of Fallot (ToF), the most common cyanotic congenital heart disease (CHD), can be associated with various genetic syndromes [1,2]. The most frequent ones are del22q11 deletion (Di George) syndrome and Down syndrome (DS) in 15% and 7% of cases, respectively, followed by Noonan, Holt-Oram, and VACTERL syndromes [1,2,3,4,5,6,7]. Previously, an association has been reported between the presence of a genetic syndrome in patients with ToF and additional risks to the primary repair [8,9,10,11,12] due to the presence of associated anatomical abnormalities, immunodeficiency, or anomalies of pulmonary vascular resistance [10,11,12]. In fact, it is well established in the literature that DS patients, even those with a structurally normal heart, have a greater risk of developing pulmonary arterial hypertension and are more susceptible to common cardiovascular risk factors such as sedentary lifestyle, obesity, and hypertension [13,14]. On the contrary, del22q11 is characterized by a wide range of features, whose different expressions in the individual patient can significantly modify the clinical picture and outcome. This is even more true for cardiac pathologies, which in most cases include ToF with associated pulmonary valve atresia, interrupted aortic arch type B, truncus arteriosus, relatively important involvement of the pulmonary arteries up to major aortopulmonary collaterals, and absence of a pulmonary artery [4,15].

Therefore, in addition to the different perioperative outcomes, syndromic patients with ToF could have a worse long-term outcome when compared to non-syndromic ones [8,9,10,11]. It is possible that the consequences of ToF repair due to significant chronic pulmonary regurgitation (PR) are worse in syndromic patients. The fact that rToF patients may have compromised right and left ventricle function in the long-term, which is associated with major incidence of fatal arrhythmias, has been well documented in the literature [16]. Thus, it should be interesting to investigate if syndromic rToF patients have worse EF of both the ventricles.

This would mean that despite similar clinical characteristics, syndromic patients could have greater right ventricular (RV) dilation, which could lead to worse RV or left ventricular (LV) dysfunction, worse exercise capacity, and more frequent ventricular arrhythmic events. Consequently, they could require considerable attention and shorter time interval between each follow-up visit and be submitted to pulmonary valve replacement (PVR) more frequently than non-syndromic patients. This may be even more true in DS, as proposed by Al-Biltagi et al. [13,14], who suggested that DS children could have silent disturbed cardiac functions despite an apparently normal heart.

Despite the interesting and useful nature of this topic, little is known about the impact of the most common syndromes on the long-term follow-up after rToF [9,17,18,19]. Recently, Sullivan et al. have shown that DS is associated with a higher degree of PR and earlier PVR [19]. However, all the studies on this issue have failed to compare rToF patients with similar characteristics (anatomy, date of birth, age, surgical history, body surface area) [18,19]. Furthermore, the non-selective inclusion of patients with and without additional pathologies (i.e., complex pulmonary arterial anatomy) could lead to incorrect assessment of the possible differences between syndromic and non-syndromic patients [18,19].

Therefore, this study aims to compare two groups of rToF patients (syndromic vs. non-syndromic) with similar clinical and surgical characteristics to evaluate the impact of the different associated syndromes on cardiac magnetic resonance imaging (cMRI) data, with particular attention to the ejection fraction of both ventricles, QRS duration and adverse cardiac events. In addition, we sought to highlight the main differences between the two most represented syndromes in rToF: DS and del22q11.2. given that these are the most frequent syndromes associated with ToF.

## 2. Methods

### 2.1. Study Design and Population

This is a multicentric (Bambino Gesù Children Hospital—Rome, Adult Congenital Heart Disease Unit, Monaldi Hospital, Naples and Azienda Ospedaliero-Universitaria Ospedali Riuniti Ancona “Umberto I, G. M. Lancisi, G. Salesi”, Ancona, Italy), cohort-matched retrospective study, evaluating the differences in clinical, surgical, MRI, Holter monitor and ECG data between syndromic and non-syndromic rToF patients following the repair. We selected all syndromic rToF patients who were able to perform a cMRI examination without general anesthesia (to avoid the well-known side effects of some anesthetic medications on the ejection fraction of both ventricles) between May 2010 and August 2020 and who could be matched for date of birth, age, sex, body surface area (BSA), surgical approach, and date of cMRI with non-syndromic rToF ones. For each syndromic patient with ToF, we have selected a non-syndromic patient of the same sex and BSA, born in the same year, who had the similar surgical approach in the same period, having had the cMRI done in the same year. Therefore, they had the same follow-up duration after corrective intervention and cMRI. We excluded ToF patients with pulmonary atresia, absent pulmonary valve, double outlet right ventricle, pulmonary atresia with interventricular septal defect and major aortopulmonary collaterals. Moreover, we excluded all atrioventricular syndromic patients with associated pathologies impairing pulmonary physiology and/or other heart defects (i.e., atrioventricular canal defect). Patients who had undergone PVR before the cMRI were not included. Data concerning the type and timing of surgery (previous Blalock-Taussig shunt, type of repair), RV pressure estimated by echocardiography, Holter monitoring and ECG data were collected in addition to cMRI data. In addition, data concerning adverse cardiac events, such as sudden cardiac death, aborted sudden cardiac death, PVR, sustained and non-sustained ventricular tachycardia and New York Heart Association (NYHA) functional classification were collected. PVR was performed in patients with severe PR and symptoms attributable to volume overload, RV pressure overload or decreased exercise tolerance. In contrast, in asymptomatic patients, PVR was performed when two or more of the following cMRI parameters were present: RV end-diastolic volume indexed by BSA (RVEDVi) ≥ 150 mL/m^2^; RV end-systolic volume indexed by BSA (RVESVi) ≥ 80 mL/m^2^; RV ejection fraction (RVEF) ≤ 47%; pulmonary regurgitation fraction (PRF) ≥ 40%; LV ejection fraction (LVEF) ≤ 55%; moderate or greater tricuspid regurgitation; RV outflow tract obstruction with RV systolic pressure ≥ 0.7 systemic [20]. From 2019 onwards, following the new guidelines for congenital heart disease patients, we decided to perform PVR for RVEDVi values ≥ 160 mL/m^2^ [21,22]. Finally, the comorbidities known to be more frequently associated with syndromic patients, such as diabetes, thyroid disorders, arterial hypertension and obesity, were evaluated. All patients included gave written informed consent, in agreement with the Declaration of Helsinki. Even though syndromic patients selected to perform cMRI without anesthesia must have had a mild intellectual disability, otherwise they would not have collaborated when asked to maintain the voluntary apnea required to acquire the images in both paediatric and adult patients, informed consent was given in most cases by a parent and/or the legal tutor for underage participants. The ethics committee approved this study (Prot. number 341/2015). 

### 2.2. cMRI Exams

A 1.5 T scanner (at Bambino Gesù Children’s Hospital, we used an Achieva 1.5 T scanner, Philips Medical, Best, The Netherlands, up to 2014, and an AERA 1.5 T scanner, Siemens, Erlangen, Germany afterward; an Achieva 1.5 T scanner, Philips Medical, Best, The Netherlands was used in Naples and a Signa Hdx, General Electric Healthcare, Milwaukee, Wisconsin in Ancona) was used to perform the cMRI examinations, following a study protocol for patients with rToF, as suggested by the literature [23,24]. The scanner includes cine steady-state free precession sequences to assess volume and function, multiple sequences to assess anatomy, and phase-contrast imaging to measure flow at the pulmonary, aortic valve and both pulmonary arteries. 

### 2.3. Image Analysis

The imaging data collected were analyzed offline on a separate workstation using a cardiac post-processing software (Viewforum, Philips Medical, Best, The Netherlands, CMR42, Circle Cardiovascular Imaging, Calgary, AB, Canada). LV and RV volumes were measured via manual segmentation of the endocardial border of both ventricles on short-axis cine images at end-diastole and end-systole, which was subsequently calculated using the method of discs [25]. Papillary muscles and trabeculations were considered part of the ventricular cavity [26]. EF was calculated from the volumes assessed. All volumes were indexed to BSA, calculated using the formula of DuBois et al. [BSA (m^2^) = 0.007184 × Height (cm) 0.725 × Weight (kg) 0.425] and compared with normal values published by Kawel-Boehm et al. [27]. In each center, there was only one observer who analyzed all the exams. Inter-observer and intra-observer comparisons of cMRI quantification were not higher than 10% for volumes and EFs of both ventricles, as reported in previous papers [26,28]. A semiautomatic edge-detection algorithm with operator correction was used to calculate blood flow from phase-contrast images. The regurgitant fraction was calculated as the retrograde flow divided by the forward flow. We considered PR to be mild if the regurgitant fraction was less than 20%, moderate if between 20 and 40%, and severe if greater than 40%. 

In addition to RV pressure ≥ 45 mmHg in echocardiography, the presence of two or more of the following cMRI criteria was required to establish the diagnosis of pulmonary artery/RV outflow tract stenosis: (1) flow velocity across the RV outflow tract or a branch pulmonary artery ≥ 3 m/sec, (2) abnormal pulmonary artery size, (3) blood flow maldistribution (RPA < 40%; LPA < 20%). RV dysfunction was taken into consideration in patients with a RVEF ≤ 47%, and LV dysfunction in the presence of a LVEF ≤ 50%. 

### 2.4. Statistical Analysis

The Shapiro–Wilk test was used to assess all continuous variables for normality and for the examination of their histograms. As appropriate, data are presented as percentages and frequencies, mean ± standard deviation, or median and 25–75th percentile (Q1, Q3). Unadjusted differences were compared using the Wilcoxon Sum Rank Test or t-test, as appropriate. Either Fisher’s exact or the Chi-Square test were used to compare dissimilarities in categorical variables, as appropriate. In order to identify predictors of LV dysfunction, univariate and multivariate logistic regression models were used. We then introduced the variables that had a *p*-value < 0.1 at the univariate analysis into the multivariable logistic regression models. Afterward, the backward stepwise regression method was used to select the best fit. Subsequently, variables that did not show a statistically significant effect on the prevalence of LV dysfunction (*p* ≥ 0.05) were removed from the multivariable models. Associations between the investigated variables and the likelihood of LV dysfunction over the follow-up period were estimated using hazard ratios (HR) and their 95% confidence intervals (95% CI). The Cox proportional hazards model was applied to calculate each clinical variable’s adjusted relative hazards of outcome events. The forward stepwise regression model based on the Akaike information criterion was used to select the multivariable analysis model. Only variables with a *p*-value < 0.1 in the univariate Cox regression analysis were subsequently introduced into the multivariable Cox regression model. Only *p* values < 0.05 were considered statistically significant. All tests were two-tailed, and the analyses were performed using computer software packages (SPSS-24.0, IBM, New York, NY, USA).

## 3. Results

A total of 108 rToF patients were selected from our cohort. Fifty-four syndromic patients were matched with 54 non-syndromic ones. All characteristics of each group are reported in Table 1.

The most prevalent genetic syndromes were del22q11.2 and DS, encountered in 23 (42.6%) and 17 (31.5%) patients, respectively. In the remaining 14 patients (25.9%), the following syndromes were detected: Noonan (*n* = 5), VACTERL (*n* = 3), Goldenhar (*n* = 2), Williams (*n* = 2), Kleefstra (*n* = 1) and subterminal del 10p15.1 + dup 10p14 (*n* = 1). The latter showed hypoplastic pulmonary branches. The comorbidities found in our syndromic population were: 4 obesity, 1 hypertension and 8 thyroid dysfunctions (Table 1). Thyroid dysfunction affected mostly DS patients (7/8). Conversely, no comorbidities were found in the non-syndromic patients. Both groups were predominantly in NYHA class I, without a difference (Table 1).

**Figure 1:**
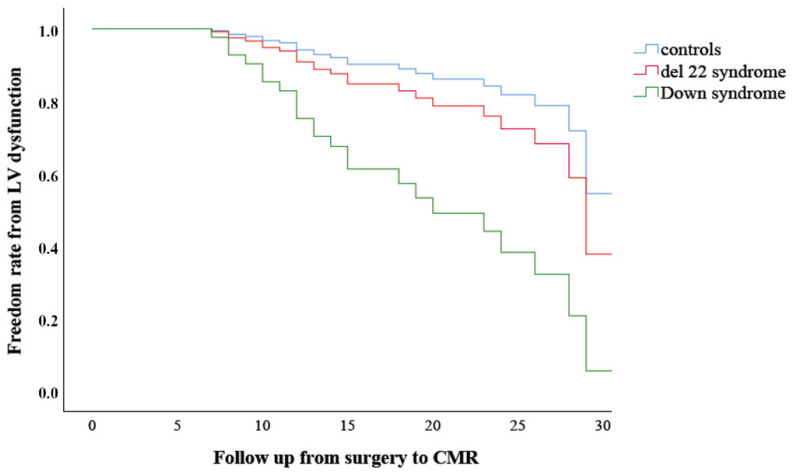
Legend. Multivariate Cox regression analysis for left ventricular (LV) dysfunction development adjusted for age at first repair.

The syndromic ToF patients were older at the repair (median age 12 vs. 7 months; *p* = 0.002) than non-syndromic ones. On the contrary, they were slightly younger at PVR (mean age 15.9 ± 5.6 vs. 19.5 ± 6.0 years, *p* = 0.049), although the percentage of PVR was similar in the two groups. Regarding the MRI parameters, we observed a major presence of LV dysfunction in the syndromic group compared to the non-syndromic one (32% vs. 15%, *p* = 0.040), besides the lower values of LVEDVi (median 74.0 mL/m^2^ vs. 80.0 mL/m^2^, *p* = 0.018) (Table 1). When comparing the two syndromes more frequently present in our cohort, DS had both a lower LVEF (50.0 ± 9.0% vs. 56.0 ± 7.0%, *p* = 0.031) and higher PRF (51.0 ± 9.0% vs. 41.0 ± 16.0%, *p* = 0.021) (Table 2). Surprisingly, the median QRS duration was significantly longer in del22q11.2 patients compared to the DS ones (median 133 ms vs. 120 ms, *p* = 0.030). There was a low incidence of adverse cardiac events during the study period (only four episodes of NS-VT). Finally, DS remained an independent prognostic factor for LV dysfunction, compared to del22q11.2 and non-syndromic patients with an HR of 5.245 (95 CI 1.709–16.100, *p* = 0.004) during a median follow-up of 14.5 years (QR 12–23) (Figure 1) on multivariate Cox regression analysis adjusted for age at first repair.

## 4. Discussion

Our study, comprising syndromic rToF patients, showed that, among the cMRI parameters usually evaluated in this cardiac disease, LV dysfunction seemed to be the only one significantly affected by the presence of a genetic syndrome. In addition, our study has documented that Down and DiGeorge syndromes, in the absence of extracardiac features, do not appear to have worse outcomes at follow-up in terms of fatal arrhythmic events, sudden death, and PVR rate. These data agree with Blais et al., who document similar long-term survival in 960 ToF patients for both the “classic” non-syndromic ToF group and “classic” ToF with genetic conditions [9]. In fact, they reported that during the first 30 years of life, the estimated number of interventions and hospitalizations stays small, and mortality before surgical correction represents approximately half of the overall mortality during the first 30 years of life, mainly in syndromic patients [9]. The importance of taking into consideration syndromic patients with “classic” ToF and comparing them with non-syndromic patients with similar anatomical characteristics at birth and a similar type of surgery is also supported by the above-mentioned study [9]. In fact, the latter has shown that the 30-year survival period varied considerably according to clinical profiles, highlighting the strong independent effects of the ToF types and genetic condition. Kauw et al. have also confirmed such data by reporting worse survival in the long-term follow-up in the group with pulmonary atresia with ventricular septal defect (PA-VSD) [29] when evaluating the difference in outcomes between del22q11.2 syndrome patients with ToF and those with PA-VSD. Therefore, we should consider the heterogeneity of the effects of genetic conditions on the risk of cardiac adverse events between ToF types.

In our cohort, the incidence of PVR was similar in both groups, but syndromic ToF patients were significantly younger at the time of the procedure (median age 15 vs. 18 yrs, *p* = 0.053). Blais et al. documented that the presence of genetic conditions could be unassociated with the number of cardiovascular interventions in rToF, except for 22q11 deletions in subjects with ToF and pulmonary atresia [9], supporting our results. On the contrary, in Sullivan et al.’s study [19], DS was associated with an increased degree of PR and an earlier PVR after ToF repair. Surprisingly, we only found a greater degree of PR in DS patients when compared to the remaining patients and the other prevalent syndrome present in our rToF population.

In our opinion, the importance of our study is represented by the fact that the presence of a genetic syndrome, particularly DS, seems to be associated with a statistically significant major possibility of developing LV dysfunction in the rToF population. Lower values of LVEF in DS patients with ToF have already been documented by Sullivan et al. [19], however, these results were not statistically significant.

The lower LVEF could be explained by the longer period of hypoxia to which the myocardium of these patients is often subjected. In fact, due to various anatomic factors, syndromic patients in our study were older at surgical repair and had a higher percentage of palliation (although not significant), as already shown by Michielon et al. [10,11]. Nevertheless, it is difficult to understand why, in a heart disease such as ToF that first involves the right chambers of the heart and only then affects the left ones, there is a further decrease in LV function before the RV in DS patients [30]. The reason for this rather surprising finding is still not entirely clear, but it could be that the presence of silent defects in cardiac function is merely a characteristic of DS patients. Indeed, LV dysfunction is associated with such syndromes, as suggested by Al-Biltagi et al., who highlighted the presence of LV diastolic dysfunction in DS children with anatomically normal hearts [14] This finding is also supported by Balli et al., who documented lower strain values of LV in the basal, mid and apical segments in a nearly 10-year study of DS children without congenital heart disease [31]. Therefore, if there is silent LV dysfunction in DS individuals without heart disease already at an early age, it is likely that it could manifest in adulthood, even more so in the presence of congenital heart disease. Prospective studies with greater sample sizes are needed to better understand these results, given that LV dysfunction represents an independent risk factor for ventricular tachycardia and all-cause mortality in rToF in the third or fourth decade of their life [32,33].

We did not find a significant difference in the QRS duration between syndromic and non-syndromic patients. On the contrary, DS patients showed shorter QRS duration compared with del22q11 patients. This could be due to the different types of surgery performed in the two groups (extension of ventriculotomy, enlargement of the pulmonary trunk, etc.). Finally, the incidence of arrhythmic events in our overall population was very low, and therefore, although the incidence of arrhythmic events seems not to be affected by the syndrome, we could not draw any conclusions on this matter.

## 5. Study Limitation

This study presents some limitations. Firstly, estimating the differences in transannular patch sizes in our cohort was not possible. Similarly, predicting the extent to which these patches may impact ventricular function and the degree of RV dilation, in addition to the well-known pulmonary insufficiency, is also quite challenging. Moreover, we were unable to correlate ventricular dysfunction with the possible presence and extent of delayed enhancement because this parameter was not taken into consideration, since it had not been evaluated in all patients. Finally, the small number of patients in our cohort may have limited the statistical power of the analysis by potentially failing to identify other significant associations.

## 6. Conclusions

Our study demonstrated that syndromic ToF patients could have a higher incidence of LV dysfunction than non-syndromic patients, independently from the surgical technique, stage, and age at repair evaluated by cMRI 15 years after repair. However, the incidence of adverse events in the follow-up period examined seems not to have been affected by the presence of a genetic syndrome in the absence of additional extracardiac anomalies.

## Figures and Tables

**Table 1:** Clinical, ECG and cMRI characteristics according to the presence of a genetic syndrome.

	Syndromic ToF (*n* = 54)	Non-Syndromic ToF (*n* = 54)	*p*-Value
Male sex, *n* (%)	29 (54)	29 (54)	0.999
Age at last examination (yrs) (median, IQR)	23 (17–29)	22 (17–29)	0.990
NYHA Class I, *n* (%)	49 (91)	51 (94)	0.462
Comorbidities, *n* (%)	14 (26)	0 (0)	<0.001
Age at cMRI (yrs) (median, IQR)	15 (13–24)	15 (13–24)	0.861
BSA (m^2^) (mean, standard deviation)	1.4 ± 0.3	1.5 ± 0.3	0.067
BMI at cMRI (mean, standard deviation)	22.7 ± 5.7	21 ± 3.8	0.068
Palliations, *n* (%)	13 (24)	6 (11)	0.128
Transannular patch, *n* (%)	51 (94)	45 (83)	0.066
Age at cardiac repair (months) (median, IQR)	12 (7–28)	7 (4–14)	0.002
PVR, *n* (%)	23 (43)	21 (39)	0.695
Age at PVR (yrs) (median, IQR)	15 (12–20)	18 (15–25)	0.053
Time between surgery and cMRI (yrs) (median, IQR)	15 (12–23)	15 (12–22)	0.897
RVEDVi (mL/m^2^) (median, IQR)	127 (108–157)	137 (120–155)	0.302
RVESVi (mL/m^2^) (median, IQR)	63 (48–85)	61 (53–74)	0.786
RVESVi > 80 mL/m^2^, *n* (%)	17 (32)	10 (18)	0.120
RVEF (%) (mean, standard deviation)	52 ± 7	54 ± 6	0.104
RVEF < 47%, *n* (%)	15 (28)	8 (15)	0.100
LVEDVi, (mL/m^2^) (median, IQR)	74 (67–84)	80 (74–92)	0.018
LVESVi, (mL/m^2^) (median, IQR)	33 (27–42)	35 (30–40)	0.635
LVEF (%) (mean, standard deviation)	55 ± 8	57 ± 7	0.207
LVEF < 50%, *n* (%)	17 (32)	8 (15)	0.040
PRF, *n* (%) (median, IQR)	45 (30–53)	41 (30–51)	0.347
RVOT/PA stenosis, *n* (%)	13 (24)	10 (19)	0.639
QRS, msec (median, IQR)	128 (110–148)	123 (120–149)	0.849
VE, *n* (%)	3 (6)	1 (2)	0.618

Legend Table 1: BMI: body mass index; BSA: body surface area; cMRI: cardiac magnetic resonance imaging; comorbidities: obesity, hypertension, thyroid dysfunction; LVEDVi: indexed left ventricular end-diastolic volume; LVEF: left ventricular ejection fraction; LVESVi: indexed left ventricular end-systolic volume; NYHA: New York Heart Association Functional Classification; PRF: pulmonary regurgitation fraction; PVR: pulmonary valve replacement; RVEDVi: indexed right ventricular end-diastolic volume; RVEF: right ventricular ejection fraction; RVESVi: indexed right ventricular end-systolic volume; RVOT/PA: right ventricular outflow tract/pulmonary artery; VE: ventricular events. Data are presented as frequencies and percentages, mean ± standard deviation, or median and interquartile range, as appropriate.

**Table 2:** Clinical, ECG and cMRI characteristics according to the type of syndrome.

	del22q11 Syndrome (*n* = 23)	Down Syndrome (*n* = 17)	*p*-Value
Male sex, *n* (%)	15 (65)	8 (47)	0.337
Age at last examination (yrs) (median, IQR)	21 (17–28)	25 (19–28)	0.537
NYHA Class I, *n* (%)	21(91)	15(88)	0.749
Comorbidities, *n* (%)	7(30)	8(47)	0.283
Age at cMRI (yrs) (median, IQR)	15 (12–24)	17 (14–23)	0.640
BSA (m^2^) (mean, standard deviation)	1.4 ± 0.3	1.5 ± 0.3	0.738
BMI at cMRI (mean, standard deviation)	22.3 ± 4.6	24.5 ± 6.5	0.212
Palliations, *n* (%)	5 (22)	5 (29)	0.717
Transannular patch, *n* (%)	23 (100)	16 (94)	0.239
Age at cardiac repair (months) (median, IQR)	15 (7–27)	16 (10–35)	0.556
PVR, *n* (%)	10 (43)	8 (47)	0.999
Age at PVR (yrs) (median, IQR)	12 (10–18)	17 (15–21)	0.155
Time between surgery and cMRI (yrs) (median, IQR)	14 (12–23)	15 (12–20)	0.989
RVEDVi (mL/m^2^) (median, IQR)	131 (113–151)	122 (110–175)	0.945
RVESVi (mL/m^2^) (median, IQR)	63 (51–85)	71 (48–92)	0.786
RVESVi > 80 mL/m^2^, *n* (%)	7 (30)	6 (35)	1.000
RVEF (%) (mean, standard deviation)	54 ± 7	51 ± 8	0.275
RVEF < 47%, *n* (%)	3 (13)	6 (35)	0.134
LVEDVi, (mL/m^2^) (median, IQR)	77 (68–88)	70 (62–84)	0.245
LVESVi, (mL/m^2^) (median, IQR)	33 (28–42)	34 (26–43)	0.808
LVEF (%) (mean, standard deviation)	56 ±7	51 ± 9	0.031
LVEF < 50%, *n* (%)	5 (22)	9 (53)	0.052
PRF (%) (mean, standard deviation)	41 ± 16	51 ± 9	0.021
RVOT/PA stenosis, *n* (%)	4 (17)	5 (29)	0.456
QRS, msec (median, IQR)	133 (110–158)	120 (91–129)	0.030
VE, *n* (%)	2(9)	1(6)	0.999

Legend Table 2: BMI: body mass index; BSA: body surface area; cMRI: cardiac magnetic resonance imaging; comorbidities: obesity, hypertension, thyroid dysfunction; LVEDVi: indexed left ventricular end-diastolic volume; LVEF: left ventricular ejection fraction; LVESVi: indexed left ventricular end-systolic volume; NYHA: New York Heart Association Functional Classification; PRF: pulmonary regurgitation fraction; PVR: pulmonary valve replacement; RVEDVi: indexed right ventricular end-diastolic volume; RVEF: right ventricular ejection fraction; RVESVi: indexed right ventricular end-systolic volume; RVOT/PA: right ventricular outflow tract/pulmonary artery; VE: ventricular events. Data are presented as frequencies and percentages, mean ± standard deviation, or median and interquartile range, as appropriate.

## Data Availability

Not applicable.
